# Diet and Microbiome in Health and Aging

**DOI:** 10.3390/nu14163250

**Published:** 2022-08-09

**Authors:** Silvia Arboleya, Sonia González, Nuria Salazar

**Affiliations:** 1 Department of Microbiology and Biochemistry of Dairy Products, Instituto de Productos Lácteos de Asturias (IPLA-CSIC), 33300 Villaviciosa, Spain; 2 Diet, Microbiota and Health Group, Instituto de Investigación Sanitaria del Principado de Asturias (ISPA), 33011 Oviedo, Spain; 3 Department of Functional Biology, University of Oviedo, 33006 Oviedo, Spain

After several years of research, sufficient evidence has been found supporting that diet is one of the main factors able to modulate both composition and activity of the intestinal microbiota, thus positioning it as a cornerstone in the host-microbiota interface. The gut microbiota plays a crucial role in the maintenance of normal host physiology. The rapid development of next-generation sequencing methods for nucleic acids, in the last decade, has facilitated in-depth studies of gut microbiome composition and function.

The articles collected in this Special Issue of Nutrients journal are intended to contribute to the progress of knowledge in the field as well as the basis for putative dietary interventions aimed at counteracting microbiota dysbiosis. These novel papers deal with the study of the relationship of diet on the intestinal microbiota from the early stages of life, deepening in certain pathologies, particularly relevant in this period of life, such as allergies, autism or overweight, up to adulthood and senescence. In addition, comprehensive review papers on hot topics such as the gut-brain axis, or the potential benefits of probiotics and prebiotics in the diet for allergy modulation were included. By providing updated and contrasted data, the authors propose several hypotheses that will be addressed in future research, which will undoubtedly arouse the interest of Nutrients journal readers.

The correct establishment of the gut microbiota at early life is known to be a milestone process for the later health of humans. Exponential studies during the last years have correlated aberrant gut microbiota colonization at the beginning of life with impairment on the intestinal, immune or nervous systems development [1]. Overweight, allergic diseases or neurodevelopmental disorders, like autism spectrum disorder (ASD), have been associated with gut microbiota alterations. Therefore, studying the composition of the gut microbiota at early life to be used as a predictor or to be target for modulation, is of great interest to prevent possible future diseases. In this context, Gonzalez et al. [2] evaluated the link between gut microbes and infant weight gain in the course of the first year of life in a cohort of full-term one-month aged neonates. They found significant associations between specific microbial groups and higher weight at 6 and 12 months, albeit being differently in vaginally and C-section delivered babies. Those gut microbes could be considered as potential microbial predictors for later weight gain.

The study of the connection between gut microbes, their metabolites and brain is currently favorable. Recent studies provide a close correlation of gut microbiota with different behavioral and cognitive traits, becoming a key stimulus during the first stages of neurodevelopment [3]. The exhaustive review by Johnson et al. [4] summarized the putative mechanisms implicated in the microbiome-brain interaction in the context of ASD. Genetic, environmental and epigenetic factors take part in an etiology puzzle that is not yet fully understood, in which the gut microbiome but also the mother’s vaginal and oral microbiomes are playing a role. The authors highlighted diet and probiotics as gut microbiome modulators promising breakthrough interventions in the direction to get more individualized treatment approaches with lower side effects to guarantee the best clinical outcomes. Atopic diseases like asthma and allergic rhinitis often begin in early childhood when intestinal microbiota is underdeveloped. Evidence clearly supports a role for gut colonization in promoting and maintaining a balanced immune response in early life, thus this period could be considered as a window of opportunity [5]. Meirlaen et al. [6] aimed in their review to investigate if prevention and/or treatment of those atopic diseases could be accomplished by targeting gut microbiome. They performed an up to date search including both animal and clinical studies where probiotics, prebiotics or synbiotics were administered for the prevention or treatment of asthma and allergic rhinitis. The authors concluded that the current evidence is not enough to make recommendations of the use in children mainly due to the large heterogeneities derived from clinical study designs, but highlighted the benefits arisen from controlled pre-clinical studies. In concordance, they pointed out the need of well-designed and standardized studies to further clarify the action of those compounds on atopic diseases.

Diet has been identified as one of the main factors influencing gut microbiota modulation from early life, with breastfeeding as the greatest influencer at this time. In adulthood, solid evidence supports that long-term diets modulate the composition of the major microbial communities inhabiting the colon [7]. However, nowadays no reliable tool for calculating the healthiest dietary pattern in terms of microbiota has been identified for different diseases or adult life stages. We have a broad understanding of the impact of diet on the gut microbiota but formulating meaningful targeted dietary strategies remains a key challenge. In this sense, the work reported by Ruiz-Saavedra et al. [8] compared people over 50 years of age in different dietary indices, widely used in the literature, evaluating their potential for predicting the composition of the intestinal microbiota together with several other indicators of inflammatory state and oxidative stress, which are of special relevance in the aging process. On the other hand, some dietary components as well as isolated foods, have also demonstrated the capacity to modulate the intestinal microbiota at different levels. In this direction, the consumption of coffee in the regular diet, in a sample of healthy people aged between 19 and 95 years, has been associated with intestinal microbiota composition by González et al. [9]. In this descriptive and observational work, novel hypotheses have been proposed for the modulation of the *Bacteroides-Prevotella-Porphyromonas* group, associated in several studies with improved metabolic health, through coffee or the polyphenols contained in this beverage.

The impact of functional foods including probiotics, prebiotics and other bioactive compounds in the gut microbiota and host health has been evaluated in this Special issue through two *in vivo* murine models and a clinical trial. Massot-Cladera et al. [10] demonstrated that multivitamin and mineral supplementation together with prebiotic fibers (inulin and acacia gum) for 4 weeks differentially modulated gut microbiota composition, mineral absorption, and some immune and metabolic biomarkers in Wistar adult rats. Intestinal immune enhancement was reported in inulin-enriched supplement whereas acacia fiber supplement had stronger prebiotic activity, which may favor increasing mineral absorption. In another preclinical trial employing also diabetic type 2 Wistar rats, Toejing et al. [11] assessed the potential antidiabetic properties of the strain *Lactobacillus paracasei* HII01 isolated from the fermentation of northern Thai pickle. The strain was tested alone or in combination with the first-line drug antidiabetic drug metformin during a 12 weeks’ period and potential beneficial effects were observed. The authors demonstrated that *L. paracasei* HII01 enhanced glycemic parameters including improvement in glucose intolerance, insulin, leptin and lipids levels, insulin-signaling proteins including from skeletal muscle that are involved in insulin-stimulated glucose uptake. This strain in diabetic rats also modulated the rat’s gut microbiota reducing the plasma endotoxemia and systemic inflammation and increased caecum short chain fatty acids levels. The results suggested that there were no synergistic effects of metformin and probiotic *L. paracasei* HII01 but the data pointed out that this strain could be considered as a complementary supplement dietary strategy for type 2 diabetic patients.

The importance of the correct vaginal microbiota composition in vaginal health and success in pregnancy was also assessed by Fernández et al. [12]. The authors reported differences in vaginal parameters (pH, Nugent score, microbiota composition and soluble immune factor levels) between women with reproductive failure and fertile women. The lowest vaginal pH values and Nugent scores were associated with vaginal communities dominated by lactobacilli, while those with the highest pH values and Nugent scores were associated with a depletion of lactobacilli. Moreover, for the first time an antibiotic-associated depletion of vaginal lactobacilli was associated with long-term health, infertility and lower pregnancy success rates. The administration of the strain *Ligilactobacillus salivarius* CECT5713 for 6 months was also tested by the first time to women with reproductive failure and resulted in improved reproductive success by the modulation of the gut microbiota and it also induced several changes in biochemical and immunological parameters in women who got pregnant. These results demonstrated that the assessment of the microbial profiles in the reproductive tract should be evaluated in cases of reproductive failure of unknown cause or origin and the administration of *L. salivarus* CECT5713 is a novel and promising strategy to modulate the reproductive tract microbiome in order to increase the success of pregnancy. Moles et al. [13] in a comprehensive review, also assessed the dietary changes across human history and the evolution of the gut microbiota as result to these changes. They disclose the power of diet over one-off treatments, such as probiotics or prebiotics, on the gut microbiota modulation and highlighted the need to unravel the diet-host-microbiota interaction to achieve a preventive and personalized medicine.

Demographic aging is a global challenge. Through its impact on various levels such as the immune system, digestive tract or cognitive impairment, the intestinal microbiota is a potential target for enhancing life quality throughout old age. With this aim, van Soest et al. [14] have studied the effect of the administration of a Mediterranean diet, rich in fresh fruits and vegetables, on inflammatory status and cognitive decline in European individuals over 65 years of age belonging to the NU-AGE cohort. While confirming a positive association between the consumption of a pro-inflammatory diet rich in animal products with a more pro-inflammatory microbiota, no impact on the cognitive decline of the participants was observed. Undoubtedly, slowing down cognitive decline along with the prevention of conditions such as Alzheimer’s disease is one of the major challenges of the nutrition field in the elderly in the last decade. The comprehensive review by Megur et al. [15] analyzed clinical and experimental studies highlighting the key role of gut microbiota dysbiosis in the development of Alzheimer’s disease. Several mechanisms of action are proposed through which the microbiota could act as a communicator between the gut and the brain.

In another study, the supplementation of isolated polyphenol rich fractions from blueberry (BB) employing in vitro fecal batch fermentations demonstrated the differential effects of the blueberry ingredients on the fecal microbiota composition in the artificial colon model [16]. Moreover, the same authors in a pilot clinical study reported that freeze-dried whole BB consumption by healthy female volunteers in two age groups (young and older) for 6 weeks changed the gut microbiota composition. The BB consumption produced higher effects in microbiota diversity in older women and its modulation was associated with antioxidant activity in healthy adults. These results support the idea that BB consumption is related with beneficial effects by both the polyphenolic and fiber content of this fruit and could be potentially used for a healthy ageing.

Some recent research has also suggested that physical activity, independent of diet, may impact positively on the composition of the microbiome, however this is not yet elucidated at the extremes of life. On the basis of evidence indicating that physically active seniors had better gastrointestinal health [17]. Fart et al. [18] explored gut microbiota composition and diversity in elderly people, according to their physical activity. Results showed significant reductions in the proportion of some microorganisms such as *Parasutterella excrementihominis* and *Bilophila wadsworthia* associated with a beneficial effect on gastrointestinal health.

The collection of articles included in this Special Issue evidenced some of the current progress on the knowledge about the effects of diet on host health through the gut microbiota modulation. Understanding the complex and dynamic interaction between dietary exposures and gut microbiota throughout lifespan can help to elucidate their potential role in different pathologies and to guide future strategies for the prevention and treatment of diseases.
